# Supplementary material for: Text classification models for the automatic detection of nonmedical prescription medication use from social media
Source: BMC Med Inform Decis Mak. 2021 Jan 26;21:27. doi: 10.1186/s12911-021-01394-0 (PMC7835447; doi:10.1186/s12911-021-01394-0)
Supplement: Supplementary file 1 — Additional file 1: Table 1. Categories, generic and brand names for medications included in the study. Table 2. Example Tweets for each of the four classes. Table 3. Hyper-parameters and other settings for the different classifiers. [file 12911_2021_1394_MOESM1_ESM.docx]

**Text Classification Models for the Automatic Detection of Nonmedical Prescription Medication Use from Social Media**

**Mohammed Ali Al-Garadi^1,*^, Yuan-Chi Yang^1^, Haitao Cai^2^, Yucheng Ruan^3^, Karen O’Connor^2^, Graciela, Gonzalez-Hernandez^2^, Jeanmarie Perrone^4^, Abeed Sarker^1,5^**

^1^Department of Biomedical Informatics, School of Medicine, Emory University, Atlanta, Georgia 30322, USA;

^2^Department of Biostatistics, Epidemiology and Informatics, Perelman School of Medicine, University of Pennsylvania, Philadelphia, Pennsylvania 19104, USA;

^3^School of Engineering and Applied Science, University of Pennsylvania, Philadelphia, Pennsylvania 19104, USA;

^4^Department of Emergency Medicine, Perelman School of Medicine, University of Pennsylvania, Philadelphia, Pennsylvania 19104, USA;

^5^Department of Biomedical Engineering, Georgia Institute of Technology and Emory University, Atlanta, Georgia, 30322, USA

*Corresponding Author:

Mohammed Ali Al-Garadi, PhD

101 Woodruff Circle

Atlanta, GA 30322

[maalgar@emory.edu.edu](mailto:maalgar@emory.edu.edu)

**Supplementary material**

Table 1. Categories, generic and brand names for medications included in the study. (1)

| Drug category | Generic name | Brand Name(s) |
| --- | --- | --- |
| Opioids | Oxycodone | Oxycontin, Percocet |
|  | Methadone | Dolophine |
|  | Morphine | Avinza |
|  | Tramadol | Conzip |
|  | Hydrocodone | Vicodin, Zohydro |
|  | Buprenorphine/naloxone | Suboxone |
| Benzodiazepines | Diazepam | Valium |
|  | Alprazolam | Xanax |
|  | Clonazepam | Klonopin |
|  | Lorazepam | Ativan |
| Atypical antipsychotics | Olanzapine | Zyprexa |
|  | Risperidone | Risperdal |
|  | Aripiprazole | Abilify |
|  | Asenapine | Saphris |
|  | Quetiapine | Seroquel |
| Central nervous system stimulants | Amphetamine mixed salts | Adderall |
|  | Lisdexamfetamine | Vyvanse |
|  | Methylphenidate | Ritalin |
| GABA^a^ analogs | Gabapentin | Neurontin |
|  | Pregabalin | Lyrica |

Table 2. Example Tweets for each of the four classes.

| Class | Example Tweets |
| --- | --- |
| Abuse | 1-tramadol and coffee for breakfast because i like to mix my chemicals before i drive.  2-waay too much codeine and adderal |
| Non-abuse consumption | 1- just got that valium though. about to sleep good  2- i've got the tramadol itches |
| Mention only | 1-<user> <user> put the valium down, you'll be fine  2-<user> i will join de fight against tramadol |
| Unrelated | 1- i would join lyrica but i'd cry.  2- it's hard mixing family and business, lyrica needs to work with someone else stop complaining |

Table 3: Hyper-parameters and other settings for the different classifiers.

| Algorithms | Hyper-parameters |
| --- | --- |
| CNN | We used CNN with Twitter GloVe word embedding for our multiclass PM abuse classification with the following configuration: embedding dimension=200, batch size=128, optimizer=Adam, dropout=0.2, sequence length=128, and train epochs=30. |
| Char-CNN | We used Char-CNN for our multiclass PM abuse classification with the following configuration: embedding dimension=200, batch size=128, maxlen=1024, optimizer=Adam, dropout=0.5, sequence length=128, and train epochs=30. |
| Bilstm | We used BiLSTM with Twitter GloVe word embeddings with the following configuration: embedding dimension=200, batch size=128, optimizer=Adam, dropout=0.2, sequence length=128, and train epochs=40. |
| BERT_base | The fine-tuning configuration of the BERT base in this study is as follows: BERT-base-uncased, maximum sequence length=128, train batch size=16, learning rate=2e-5, and train epochs=3. |
| BERT_large | The fine-tuning configuration of BERT-large in this study is as follows: BERT-large-uncased maximum sequence length=128, train batch size=16, learning rate=2e-5, and train epochs=3. |
| RoBERTa large | The fine-tuning configuration of RoBERTa in this study is as follows: RoBERTa-large, maximum sequence length=128, train batch size=16, learning rate=2e5, and train epochs=3. |
| AlBERT xxlarge | The fine-tuning configuration of AlBERT in this study is as follows: AlBERT xxlarge, maximum sequence length=128, train batch size=16, learning rate=2e-5, and train epochs=3. |
| XLNet | The fine-tuning configuration of XLNet in this study is as follows: XLNet-base, maximum sequence length=128, train batch size=16, learning rate=2e-5, and train epochs=3. |
| DistilBERT | The fine-tuning configuration of DistilBERT in this study is as follows: DistilBERT, maximum sequence length=128, train batch size=16, learning rate=2e-5, and train epochs=3. |

**References**

1. O’Connor K, Sarker A, Perrone J, Gonzalez Hernandez G. Promoting Reproducible Research for Characterizing Nonmedical Use of Medications Through Data Annotation: Description of a Twitter Corpus and Guidelines. J Med Internet Res. 2020 Feb;22(2):e15861.
